# Denoising very low-field magnetic resonance images using native noise modeling

**DOI:** 10.3389/fnimg.2025.1501801

**Published:** 2025-05-06

**Authors:** Tonny Ssentamu, Alvin Kimbowa, Ronald Omoding, Edgar Atamba, Pius K. Mukwaya, George W. Jjuuko, Sairam Geethanath

**Affiliations:** ^1^Department of Physiology, Makerere University, Kampala, Uganda; ^2^School of Immunology and Microbial Sciences, King's College London, London, United Kingdom; ^3^School of Biomedical Engineering, University of British Columbia, Vancouver, BC, Canada; ^4^Department Electrical and Computer Engineering, Makerere University, Kampala, Uganda; ^5^Department of Information Engineering, Università Politecnica Delle Marche, Ancona, Italy; ^6^Department of Information Engineering, University of Pisa (UNIPI), Pisa, Italy; ^7^Icahn School of Medicine at Mount Sinai, New York, NY, United States; ^8^Accessible MR Laboratory, Johns Hopkins University School of Medicine, Baltimore, MD, United States

**Keywords:** low-field MRI, MRI, denoising, native noise, deep learning (DL)

## Abstract

Low-field MRI is gaining interest, especially in low-resource settings, due to its low cost, portability, small footprint, and low power consumption. However, it suffers from significant noise, limiting its clinical utility. This study introduces native noise denoising (NND), which leverages the inherent noise characteristics of the acquired low-field data. By obtaining the noise characteristics from corner patches of low-field images, we iteratively added similar noise to high-field images to create a paired noisy-clean dataset. A U-Net based denoising autoencoder was trained on this dataset and evaluated on three low-field datasets: the M4Raw dataset (0.3T), *in vivo* brain MRI (0.05T), and phantom images (0.05T). The NND approach demonstrated improvements in signal-to-noise ratio (SNR) of 32.76%, 19.02%, and 8.16% across the M4Raw, *in vivo* and phantom datasets, respectively. Qualitative assessments, including difference maps, line intensity plots, and effective receptive fields, suggested that NND preserves structural details and edges compared to random noise denoising (RND), indicating potential enhancements in visual quality. This substantial improvement in low-field imaging quality addresses the fundamental challenge of diagnostic confidence in resource-constrained settings. By mitigating the primary technical limitation of these systems, our approach expands the clinical utility of low-field MRI scanners, potentially facilitating broader access to diagnostic imaging across resource-limited healthcare environments globally.

## 1 Introduction

Magnetic resonance imaging (MRI) is a critical component of modern medicine, contributing considerably to advances in both fundamental research and clinical patient treatment. However, over 85% of the MRI on the market are high-field (HF) scanners (*B*_0_≥1.5*T*) which are relatively expensive, occupy large footprints, and consume high power, rendering them inaccessible especially in low-income settings (Anazodo et al., 2023). This has drawn a renewed interest in low-field (LF) MRI, operating at magnetic fields (*B*_0_ < 1*T*), owing to their low cost, portability, small footprint, and low power consumption. However, LF MRI scanners suffer significantly from reduced signal-to-noise ratio (SNR) per unit scanning time (Arnold et al., 2023; Marques et al., 2019; Geethanath and Vaughan, 2019). Achieving higher SNR on such scanners requires longer scan times constrained by patient tolerance and clinical needs (Marques et al., 2019). Consequently, images acquired with low-field (LF) MRI are typically low-resolution, with limited clinical utility (Arnold et al., 2023). There is, thus, a need to develop mechanisms to improve the image quality of LF MRI while maintaining their affordability and clinical feasibility.

One approach to improve the SNR of LF MRI images is to reduce the noise using image-denoising methods. Traditionally, simple linear Gaussian and average filters, and non-linear median and bilateral filters have been used to denoise brain MRI images (Saladi and Prabha, 2017; Suhas and Venugopal, 2017; Shedbalkar et al., 2021). Some approaches adopt more involved algorithms such as adaptive filters, k-SVD, non-local means, anisotropic diffusion, and PCA-based methods to improve denoising performance (Tong et al., 2016; Saladi and Prabha, 2017). Despite their robustness, traditional approaches heavily rely on manually selected hyperparameters, which results in suboptimal denoising models, and often require re-optimization at test time, which is time-consuming (Zhang et al., 2017).

**Figure 1:**
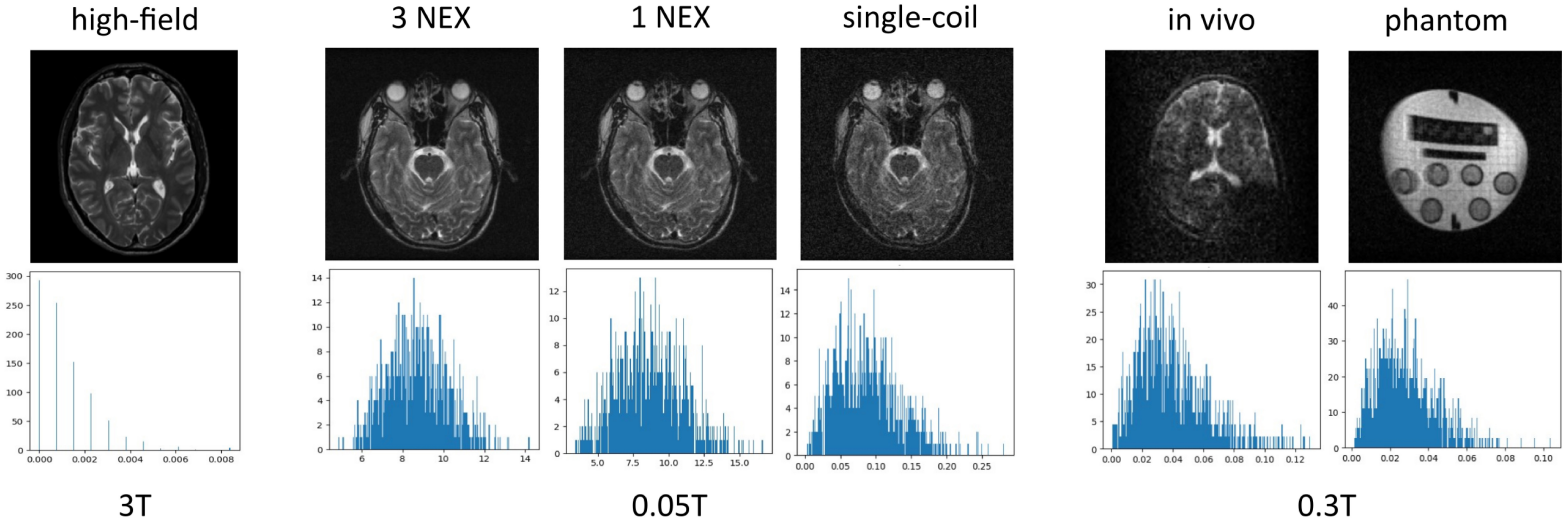
Noise characteristics in MRI images across different field strengths: the top row displays brain MRI images, while the bottom row shows corresponding histograms of pixel intensities from four corner patches. From left to right: First column shows a high-field (3T) image with minimal noise. Second and third columns display multi-channel acquisitions at 0.3T using 3 NEX and 1 NEX respectively, both exhibiting Gaussian-like noise distributions. Fourth column presents a single-coil acquisition at 0.3T, demonstrating a shift toward Rician distribution. Fifth and sixth columns show *in vivo* and phantom images at very low field (0.05T), both following Rician distributions. This comparison illustrates how noise characteristics vary with field strength, acquisition method, and number of excitations in MRI.

Over the past two decades, deep learning approaches have been proposed for LF MRI denoising, outperforming traditional methods (El-Shafai et al., 2023; Lee et al., 2017). These approaches require a paired dataset of noisy and corresponding clean MRI images to be trained. However, given that over 85% of the MRI scanners on the market are HF scanners, there is a lack of LF MRI, and even more scarce paired LF and HF MRI data (Anazodo et al., 2023). As a result, various proposed LF denoising approaches rely on simulating noisy LF data from the abundant open-source HF data (Le et al., 2021; Vega et al., 2023). These approaches generally simulate random noise following a Gaussian distribution with the expectation that this noise is representative of the noise in the LF images. However, noise in LF MRI follows a Rician distribution, as shown in Figure 1, and models trained on LF images simulated with Gaussian noise are bound to achieve suboptimal performance (Gudbjartsson and Patz, 1995). In this approach, the noise is obtained directly from the low-field MRI images and iteratively added to the high-field data to generate a training dataset to train the NNDnet. Their approach achieves a PSNR of over 38dB on the simulated data and image entropy greater than 4.25 on actual 0.36T MRI images. This work shows promising performance for using native noise, however, further investigation is required to assess the performance of such native noise-denoising approaches at very low-field MRI (0.05T), for both in- and out-of-distribution images.

In this work, we extend and demonstrate native noise denoising to 0.05T by analyzing and modeling noise from a 0.05T scanner and using it to train a robust deep-learning algorithm for denoising very low-field MRI images. We evaluate the algorithms on in-distribution and out-of-distribution data and demonstrate that; (1) models trained on native noise outperform those trained on Gaussian noise, and (2) patch-wise denoising outperforms image-wise denoising.

## 2 Methods

**Figure 2:**
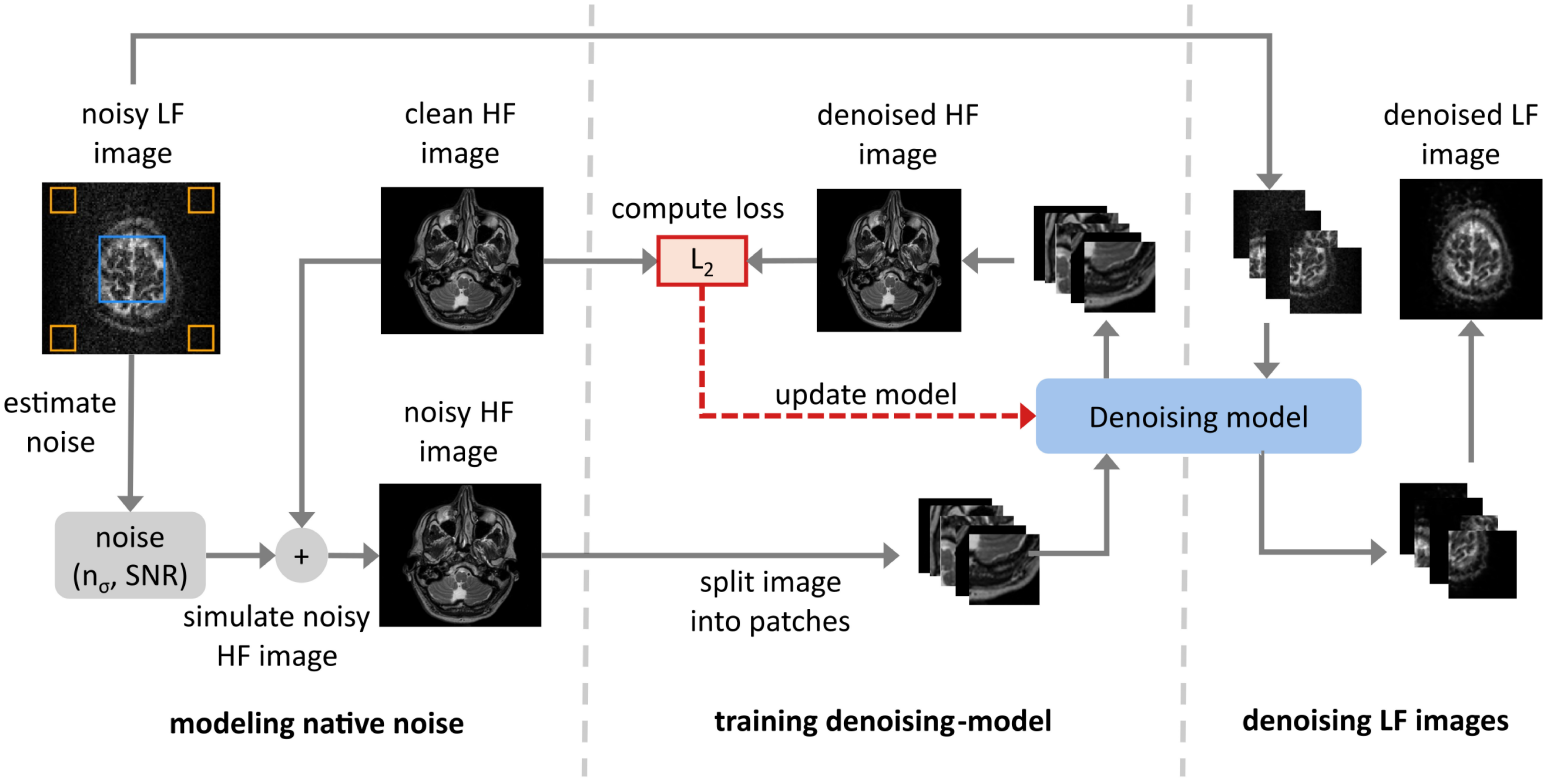
Overview of the native noise denoising approach, divided into three key stages: (a) *modeling native noise* – simulation of noise characteristics from low-field (LF) MRI images followed by adding the simulated native noise to the clean high-field (HF) MRI images to generate a noisy-clean paired dataset. (b) *training denoising-model* – the model uses the noisy-clean paired dataset to process the noisy images and attempt to recover the clean images, which are then compared to the ground truth using a loss function (c) *denoising LF images* – application of the trained model to various low-field MRI datasets, including M4Raw, *in vivo*, and phantom images, to assess its denoising performance on unseen data.

Our approach consists of three stages: noise modeling, model training, and inference, as shown in Figure 2. In the noise modeling stage, we determine the noise characteristics of the target LF MRI images and simulate this noise in HF MRI images to create a paired dataset of simulated noisy HF images and original clean HF images. During the model training stage, we employ a patch-wise approach where patches of size *N*×*N* are randomly extracted from the simulated noisy HF images and their corresponding clean HF patches. This method ensures diverse training samples, enhancing the model's robustness to various noise variations in the MRI images.

The denoising algorithm is trained on these patches to learn effective noise reduction for small image regions. In the inference stage, the entire LF image from either of the three datasets is input into the trained model for denoising, leveraging the patch-wise training to enhance overall image quality. This full-image inference approach avoids potential artifacts from stitching denoised patches together, ensuring seamless and accurate denoising.

### 2.1 Noise simulation

Noise in low-field MRI is predominantly thermal noise arising from the resistance in the radio frequency (RF) coils, with the body noise being negligible (Koonjoo et al., 2021). This noise follows a Gaussian distribution in the real and imaginary components of LF MRI images due to the linearity and orthogonality of the Fourier transform (Chaudhari and Kulkarni, 2021; Gudbjartsson and Patz, 1995). However, upon non-linear transformation from a complex to a magnitude image, this noise generally adopts a Rician distribution (Chaudhari and Kulkarni, 2021; Gudbjartsson and Patz, 1995).

Low-field MRI is also affected by electromagnetic interference (EMI), which can alter the underlying noise distribution, and EMI correction remains an active area of research (Bian et al., 2024). This study focuses on reducing thermal noise and assumes minimal EMI.

#### 2.1.1 Native noise modeling

To accurately simulate Rician noise (referred to as Native noise in this work), we iteratively add random noise to the real and imaginary components of the HF complex image until the SNR of the simulated LF image matches that of the LF images of interest. We assume that adding noise in a single step might not yield accurate simulations due to potential variations in signal levels across different images in the clean dataset, even if generated by the same scanner. This iterative process allows us to closely monitor the SNR and make precise adjustments, which we believe could result in better simulations.

Determining the step size for each iteration involved experimenting with different values. This ensured that the SNR of each simulated image was within a close range of our target SNR. Additionally, we aimed to balance simulation speed, ensuring it was optimal for precise and efficient noise addition. This dynamic adjustment of step size is intended to ensure accurate and consistent noise simulation, potentially leading to simulated LF images that closely match the characteristics of real LF images.

**Figure 3:**
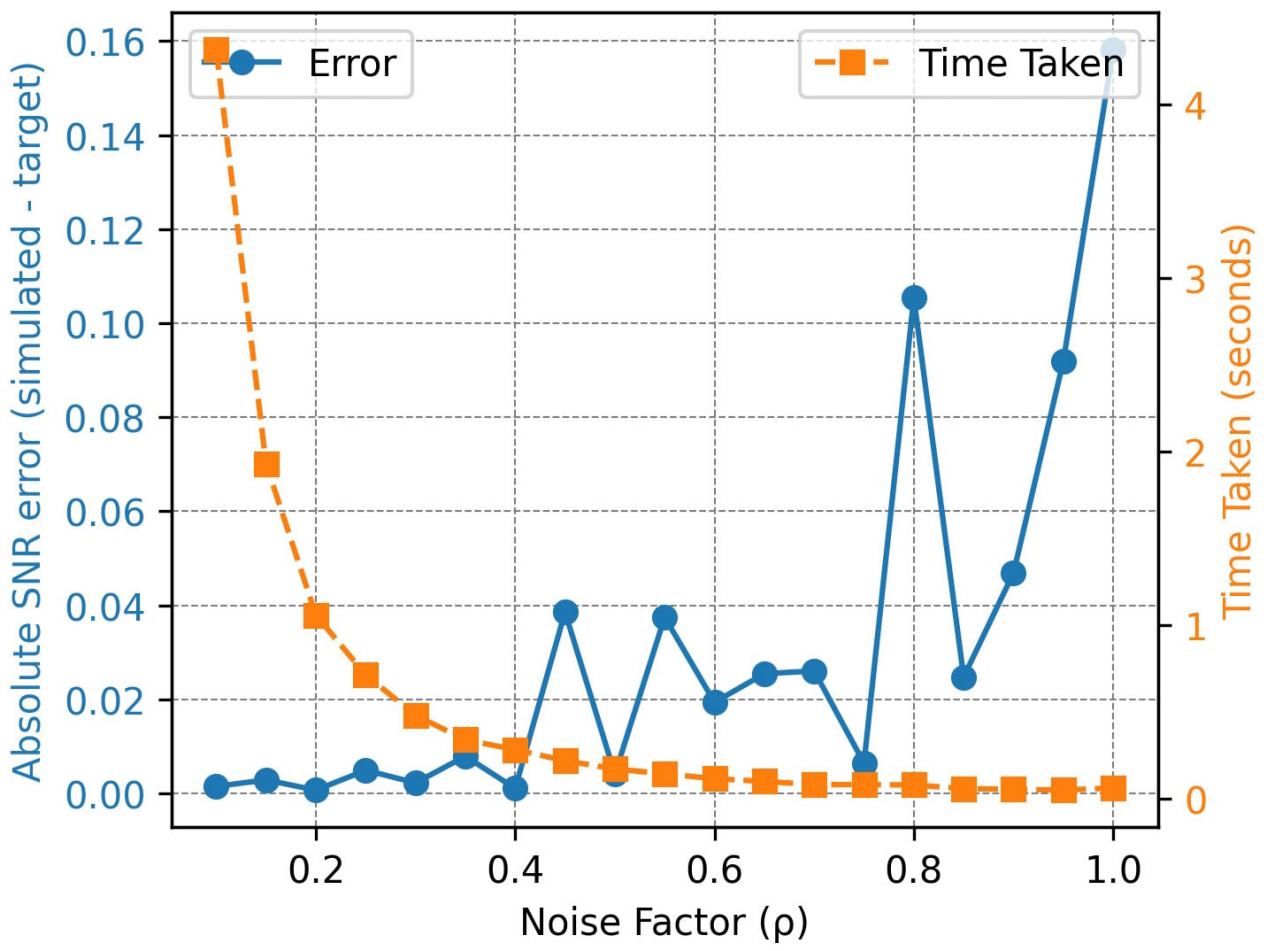
Effect of ρ on speed and accuracy for a single slice: accuracy is measured as the absolute error between the simulated SNR and the target SNR. Generally, lower values of ρ yield more accurate simulations of the target SNR but at an increased simulation time. The error curve also highlights the stochasticity of the noise simulation.

We adapt the open-source LF simulator from the Intelligent MR Framework lab for the noise simulation (Aggarwal et al., 2023). The image SNR is obtained by dividing the mean of the signal in the region of interest by the standard deviation of the noise in the corner patches (Dougherty, 2009). At each iteration, random noise, scaled by a factor ρ, is added to the complex HF image following Equations 1, 2. The choice of ρ determines the speed and accuracy of the simulated noisy images with a lower ρ yielding more accurate noise simulation but at a higher time cost as shown in Figure 3. The final simulated LF magnitude image, *Is*, is obtained from the real and imaginary components at the last iteration (Equation 3).


(1) $$\begin{array}{ll} Ir_{i} & =Ir_{i-1}+\rho \cdot n_{1_{\sigma }} \end{array}$$

(2) $$\begin{array}{ll} Im_{i} & =Im_{i-1}+\rho \cdot n_{2_{\sigma }} \end{array}$$

(3) $$\begin{array}{ll} Is_{i} & =\sqrt{Ir_{i}^{2}+Im_{i}^{2}} \end{array}$$

where *n*_1_ and *n*_2_ are drawn from zero-mean Gaussian distributions with the same standard deviation σ given by Equation 4. Two Gaussian distributions are used to ensure that the noise added to each component is independent.


(4) $$\begin{array}{l} p\left(x\right)=\frac{1}{\sqrt{2\pi \sigma^{2}}}\exp^{-\frac{x^{2}}{2\sigma^{2}}} \end{array}$$

In scenarios where only magnitude HF images are available, a complex image is approximated from the HF images with $Ir_{0}=Im_{0}=Ic/\sqrt{2}$. Note that *Ic* is scaled appropriately to ensure that it can be obtained back using the magnitude of the estimated complex image.

#### 2.1.2 Random noise modeling

To simulate Gaussian noise (referred to as random noise in this study), we add noise directly to the magnitude images. Specifically, the random noise is generated using a zero-mean normal distribution with standard deviation σ. The noise N is added to the magnitude image *I*_*m*_ to produce a noisy image $I_{m}^{\prime }$. This process can be expressed by the following equation:


(5) $$\begin{array}{l} I_{m}^{\prime }=I_{m}+N \end{array}$$

where *N* is a random noise component drawn from a zero-mean Gaussian distribution:


(6) $$\begin{array}{l} N\sim N\left(0,\sigma^{2}\right) \end{array}$$

The resulting noisy image $I_{m}^{\prime }$ obtained by directly adding the noise *N* to the original magnitude image *I*_*m*_. This method contrasts with native noise simulation, where noise is added to the real and imaginary components of the complex image before taking the magnitude. The simulated noisy data for random noise and native noise thus have different distributions. Our study compares the performance of models trained on these two types of noise to evaluate their effectiveness in denoising LF MRI images.

### 2.2 Model training

**Figure 4:**
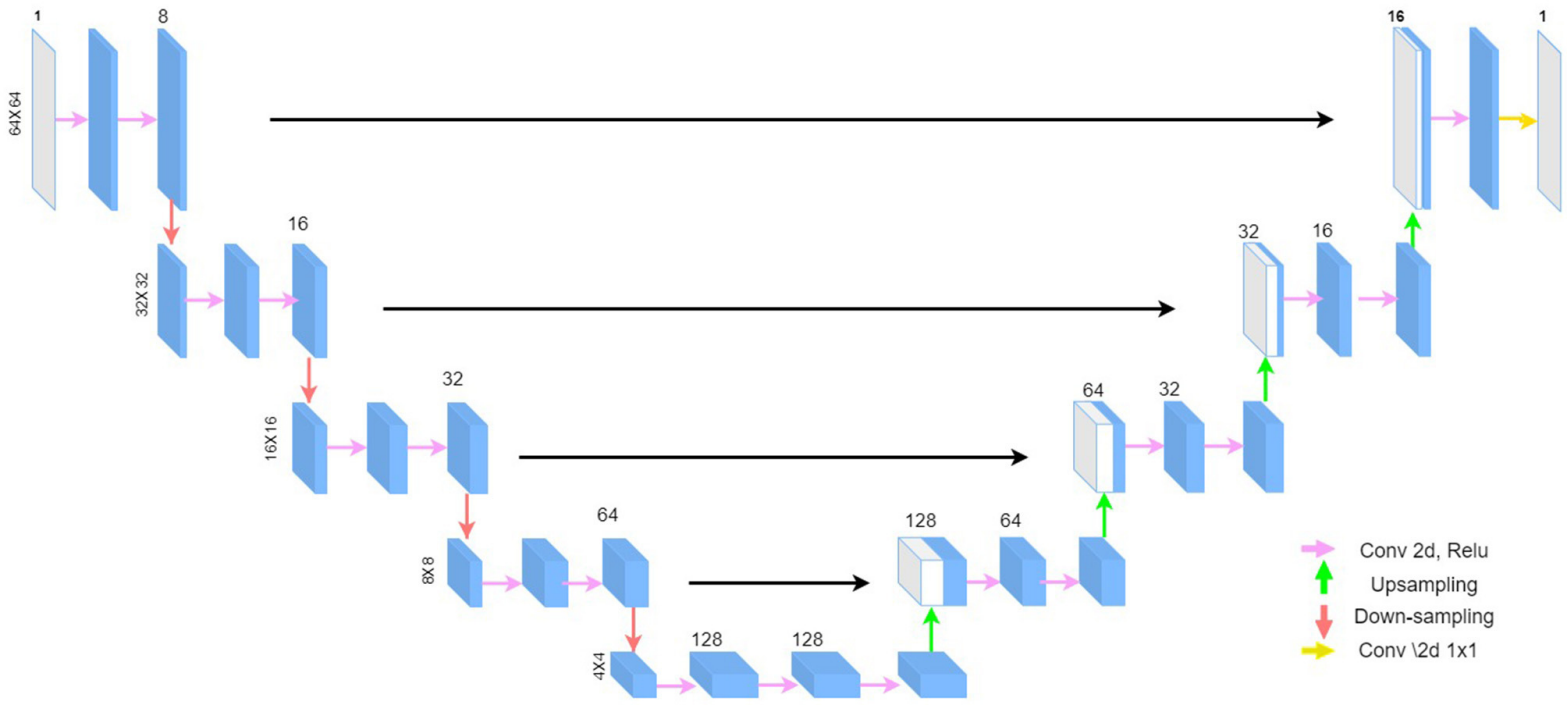
U-Net architecture: the model takes as input a square patch of size 64 × 64, extracts features through the contracting path, and reconstructs the denoised image through the expanding path. We use 8 channels in the first convolution block and double the channels after each block, similar to the original U-Net architecture.

The simulated LF data is paired with the clean HF data and used to train a denoising autoencoder to learn a non-linear mapping from the noisy images to the clean image (Goodfellow et al., 2016). Ideally, any suitable denoising autoencoder can be used, but we adopt the U-Net architecture (Ronneberger et al., 2015) originally developed for medical imaging applications and is robust for various medical image-to-image prediction tasks (Zbontar et al., 2019). The U-Net is a fully convolutional neural network consisting of a contracting path, that extracts features from the input image, and an expanding path that reconstructs the desired output from the features as shown in Figure 4.

The model has skip-connections between corresponding contracting and expanding paths to propagate high-resolution information from the contracting path to the expansion path.

We follow a patch-wise training approach, with square patches of size 64, for the model to learn the noise distribution rather than the brain structure in the HF data. The patch size was determined empirically and yielded the best results as shown in Table 1. In the U-Net architecture, the number of channels is doubled every after the max-pooling operation. The original U-Net used 64 channels in the first layer for input images of size of 572 × 572. However, since we use patches of size 64 × 64 (about 8 times smaller than 572 × 572) as inputs to the model, we correspondingly scale down the number of channels in the first convolutional block by a factor of 8.

**Table 1:** Test results: patch-wise training achieves a better SSIM on unseen HF test data across all models, regardless of the noise and dataset used.

**Datasets**	**Patch size**	**PSNR**	**SNR**	**SSIM**
**(a) Random noise denoising (RND)**				
Phantom	64	40.34	56.54	0.97
	128	40.73	57.61	0.96
	256	38.8	58.17	0.85
*In vivo*	64	32.34	54.47	0.91
	128	32.71	52.35	0.90
	256	31.29	45.99	0.64
**(b) Native noise denoising (NND)**				
Phantom	64	40.71	56.97	0.97
	128	41.39	57.00	0.97
	256	37.95	48.86	0.72
*In vivo*	64	31.57	51.55	0.91
	128	32.24	55.32	0.90
	256	31.11	54.37	0.64

Corresponding peak signal-to-noise ratio (PSNR) and signal-to-noise ratio (SNR) in dB are also included.

The model was trained on the IXI dataset which contained 1,952 image slices with dimensions 256 × 256, which yielded 1,0525 image patches of size 64 × 64. The dataset was split into 85% training, 5% validation, and 10% testing. The model was trained with the mean squared error (MSE) loss function, Adam optimizer (Kingma and Ba, 2017) with the default learning rate of 0.001, and a batch size of 64–limited by computational constraints. MSE measures pixel-wise intensity differences between the model prediction, ŷ, and the ground truth clean image, *y*, following Equation 7:


(7) $$MSE(\hat{y},y)=\frac{1}{N}\sum {\left({\hat{y}}_{i}-y_{i}\right)}^{2},$$

where *N* is the number of pixels in each image. PSNR measures the power of the maximum possible intensity in the ground truth image relative to the power of the MSE between the model prediction and ground truth, following Equation 8:


(8) $$PSNR(\hat{y},y)=10\log_{10}\frac{\max{\left(y\right)}^{2}}{MSE(\hat{y},y)}.$$

We select the model that yields the highest peak-signal-to-noise ratio (PSNR) on the validation dataset during training. We also use the structural similarity index (SSIM) to assess model performance on the high-field images. The SSIM between two images, *x* and *y*, is calculated as (Horé and Ziou, 2010):


$$SSIM\left(x,y\right)=\frac{\left(2\mu_{x}\mu_{y}+C_{1}\right)\left(2\sigma_{xy}+C_{2}\right)}{\left(\mu_{x}^{2}+\mu_{y}^{2}+C_{1}\right)\left(\sigma_{x}^{2}+\sigma_{y}^{2}+C_{2}\right)}$$

where μ_*x*_ and μ_*y*_ are the mean intensities, $\sigma_{x}^{2}$ and $\sigma_{y}^{2}$ are the variances, σ_*xy*_ is the covariance of the image patches, and *C*_1_ and *C*_2_ are constants to stabilize the division.

We developed the model using the TensorFlow framework (Abadi et al., 2015) and trained on Google Colab's Tesla T4 12GB graphical processing units (Bisong, 2019).

### 2.3 Evaluation

We used three open-source datasets in this study: the IXI brain development dataset, the 0.3T *in vivo* M4Raw dataset (Lyu et al., 2023), and a 0.05T phantom dataset. We also evaluated with a sample 0.05T *in vivo* dataset collected locally. The IXI dataset is publicly available and can be accessed at https://brain-development.org/ixi-dataset/. The dataset consists of 600 MR images collected from normal healthy subjects at three different hospitals in London using 1.5T and 3T scanners.

The data was collected for various MRI sequences but in this study, we used only the T2W images given that the low-field data available consisted of only T2W images. We randomly selected 15 volumes due to computational constraints. Each volume had about 120 slices resulting in a total of 1,812 images. Throughout the proceeding text, we refer to this data as the high-field (HF) MRI data. This data was used for training the machine learning algorithms.

The M4Raw dataset (Lyu et al., 2023) was obtained from 183 healthy volunteers using a four-channel 0.3T head coil. It comprises T1-weighted, T2-weighted, and fluid-attenuated inversion recovery (FLAIR) axial multi-repetition images.

**Figure 5:**
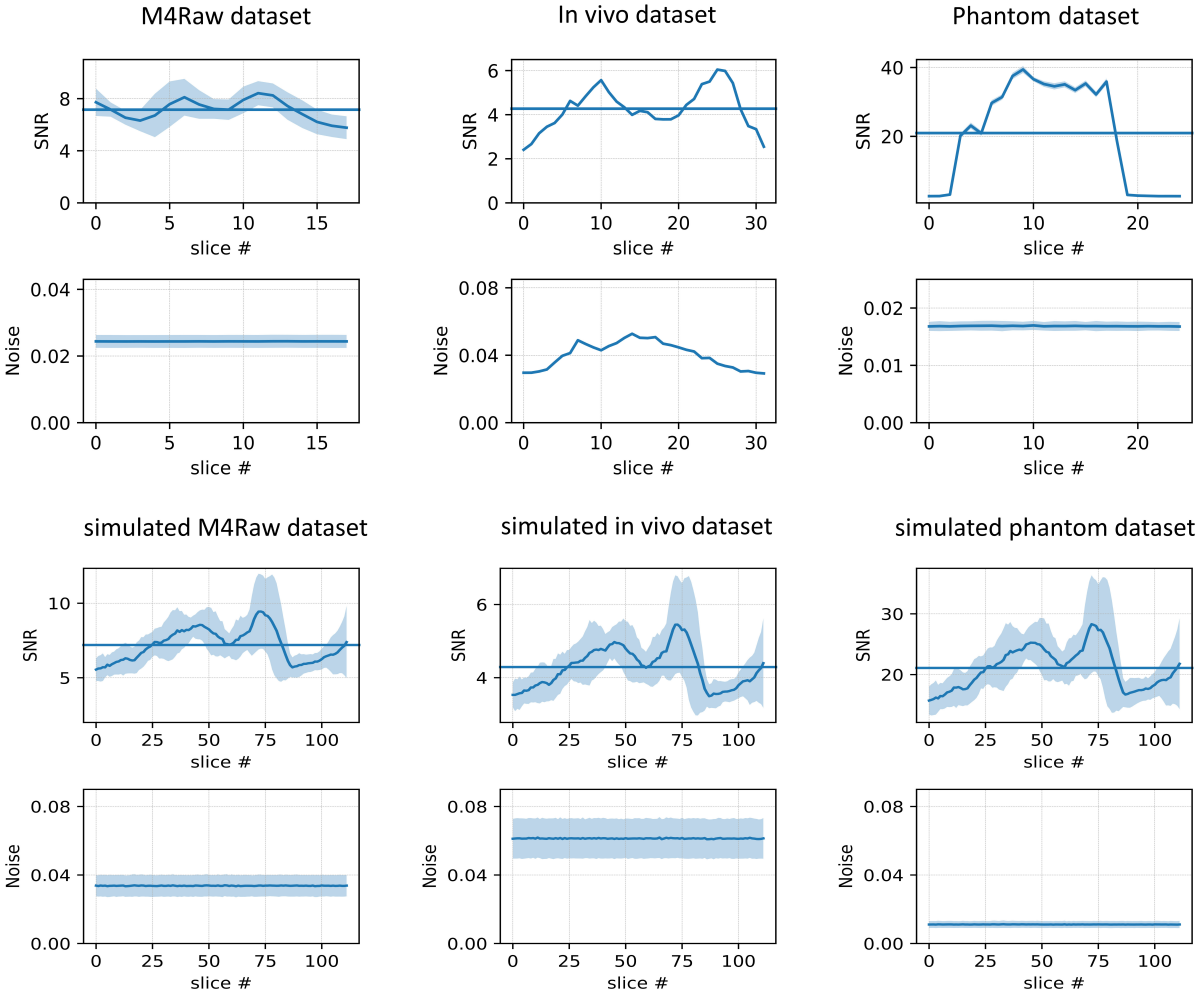
Noise simulation: this figure shows SNR and noise levels plotted as a function of slice number for three different datasets (M4Raw, *in vivo*, and Phantom), along with their respective simulated counterparts. The first row compares SNR across slices for the M4raw, *in vivo*, and phantom datasets, while the second row presents the corresponding noise levels. The third and fourth rows illustrate the same metrics as the plots above respectively for the simulated datasets. Shaded regions represent the standard deviation around the mean SNR, highlighting variability across slices. The results reveal differences in SNR trends and noise consistency between the real and simulated datasets.

In this study, we used the single-channel images to match the single-channel acquisition at low field. The single-channel images have an average SNR of 7.15 (17.06 dB) and noise standard deviation of 0.024 as shown in the first column, second row (Figure 5).

The 0.05T human brain MRI dataset was obtained from a single healthy subject, after informed consent, using the same scanner as the phantom data with a three-dimensional (3D) turbo-spin echo (TSE) with a repetition time (TR) of 2500ms and an echo time of 250ms. The data consisted of three orientations; axial, sagittal, and coronal, with a resolution of 2 × 2 × 5 mm^3^. Throughout the proceeding text, this data is referred to as the *in vivo* dataset. The data has an average SNR of 4.17 and a noise standard deviation of 0.04 as shown in the second column, second row (Figure 5).

The phantom dataset comprises T1-weighted axial Pro-MRI phantom images acquired using a single-coil 0.05T Multiwave MGNTQ MRI scanner during a repeatability study (Aggarwal P. P. K. et al., 2023). The images used were not corrected for geometric distortion. We refer to this dataset as phantom data throughout the proceeding text. The images in the phantom dataset have an average SNR of 20.96 and a noise standard deviation of 0.018 as shown in the third column, second row (Figure 5). All the LF data was used to simulate noise in the HF data.

## 3 Results and discussion

### 3.1 Effect of patch size

**Figure 6:**
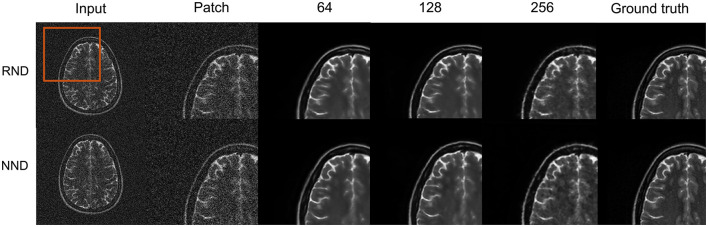
Patch-wise training: here, we show qualitative results for different patch sizes used during training. Models trained patch-wise preserve brain structure for both random noise denoising (RND) and native noise denoising (NND). The models trained on the full image (patch size of 256 × 256) distort the structure in the images as seen in the white matter regions.

Models trained using a patch-wise approach preserve brain structure while those trained on full images tend to distort brain structure as shown in Figure 6. Models trained using a patch size of 64 yielded the highest structural SSIM on the test data as seen in Table 1.

This performance can be attributed to the model's limited field of view, forcing it to learn to remove noise within a local region as opposed to learning the brain structure. It can also be noted that SSIM gradually decreases with increasing patch size.

### 3.2 Performance on LF data

The weights of the models trained with patches of 64 × 64 were frozen and evaluated on the LF datasets. We tabulate the mean (95% CI) slice-wise SNR(dB) for both methods across the different in Table 2. We performed a paired *t*-test, with a *p*-value threshold of 0.05, to compare the performance of NND and RND. Native noise denoising (NND) achieves significantly higher SNR compared to RND across all the datasets. Specifically, NND outperforms RND by 32.76%, 19.02%, and 8.16%, on the M4Raw, *in-vivo*, and phantom datasets, respectively. The NND model was also evaluated on out-of-distribution axial, coronal, and sagittal views of the *in vivo* dataset as shown in Table 3. Despite RND outperforming NND on the coronal view, NND yields consistent performance across all three views.

**Table 2:** Performance on low-field data: native noise denoising (NND) achieves higher average SNR values as compared to random noise denoising (RND) across all three datasets.

**Approach**	**M4Raw**		***In vivo***		**Phantom**	
	**SNR**	**SNR Gain**	**SNR**	**SNR Gain**	**SNR**	**SNR Gain**
RND	38.80 (38.53, 39.08)	+22.32	35.32 (32.14, 38.51)	+15.46	31.24 (30.76, 31.70)	+4.89
NND	51.51^*^ (51.31, 51.71)	+35.03	42.04* (40.49, 43.58)	+22.17	33.79* (33.27, 34.27)	+7.43
% improvement	+32.76%		+19.02%		8.16%	

^*^Indicates statistically significantly higher performance (*p* < 0.05). % improvement = (*NND*−*RND*)/*RND*.

**Table 3:** Detailed *in vivo* performance: NND exhibits consistent and reliable performance across different views of the *in vivo* data, unlike RND whose performance drastically drops for sagittal view.

**Approach**	**Axial**		**Sagittal**		**Coronal**	
	**SNR**	**SNR Gain**	**SNR**	**SNR Gain**	**SNR**	**SNR Gain**
RND	45.68 (43.73, 47.64)	+24.41	14.09 (12.53, 15.65)	–3.19	46.20* (45.16, 47.25)	+25.16
NND	46.22 (43.73, 47.64)	+24.95	38.03* (35.08, 40.99)	+20.76	41.86 (39.82, 43.89)	+20.81
% improvement	+1.18%		+170.00%		–9.39%	

^*^Indicates statistically significantly higher performance (*p* < 0.05). % improvement = (*NND*−*RND*)/*RND*.

**Figure 7:**
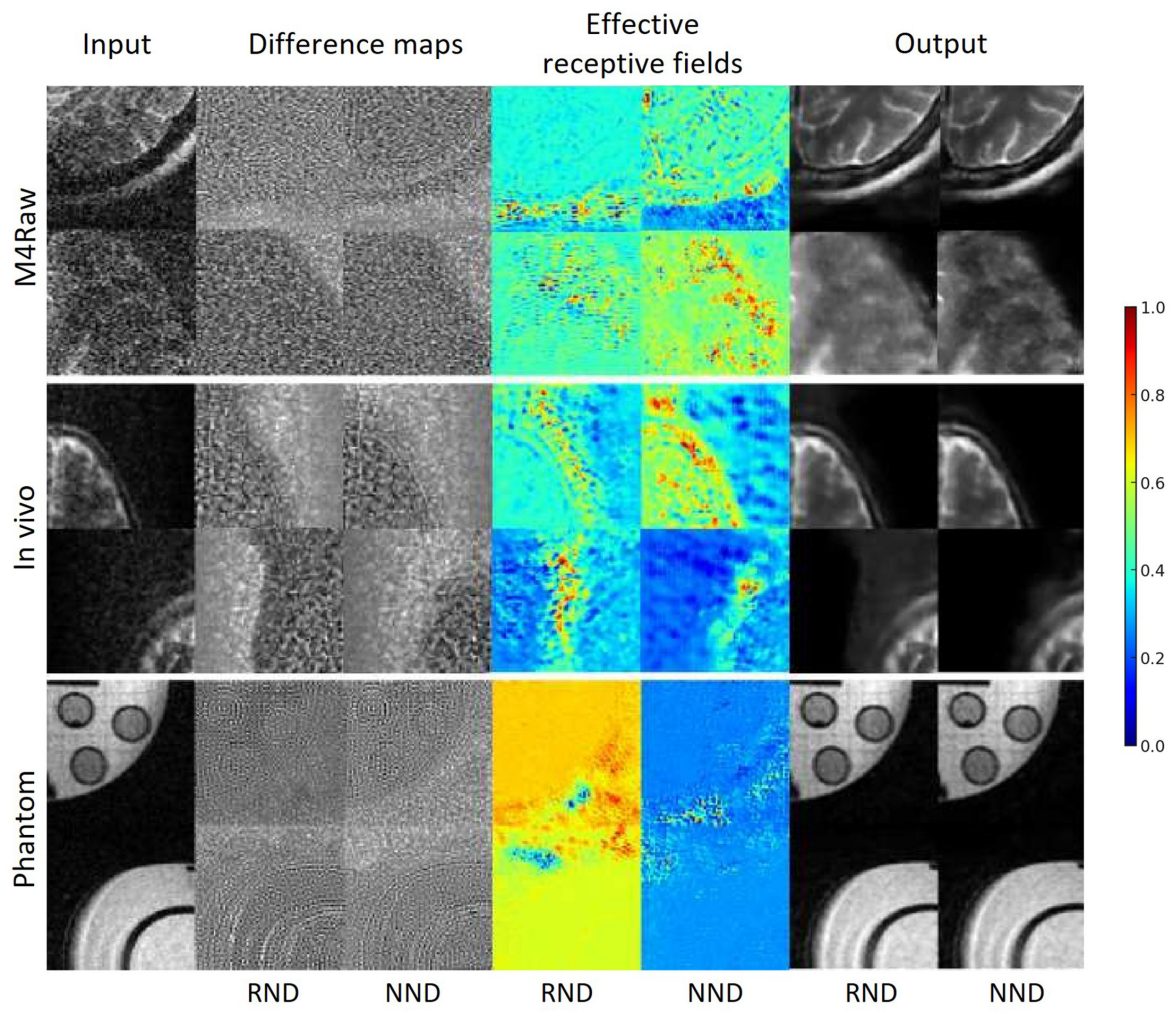
Qualitative evaluation on low-field MRI data. The figure compares denoising performance across three datasets (M4Raw, *in vivo*, Phantom). The first column shows the Input image patch, followed by difference maps for the Random Noise Denoising (RND-Diff) and Native Noise Denoising (NND-Diff). The fourth and fifth columns depict the Effective Receptive Fields (ERF) corresponding to these difference maps, with the NND model demonstrating structural preservation, particularly around edges and the region of interest. The color scale reflects activation intensity, from high (red) to low (blue).

**Figure 8:**
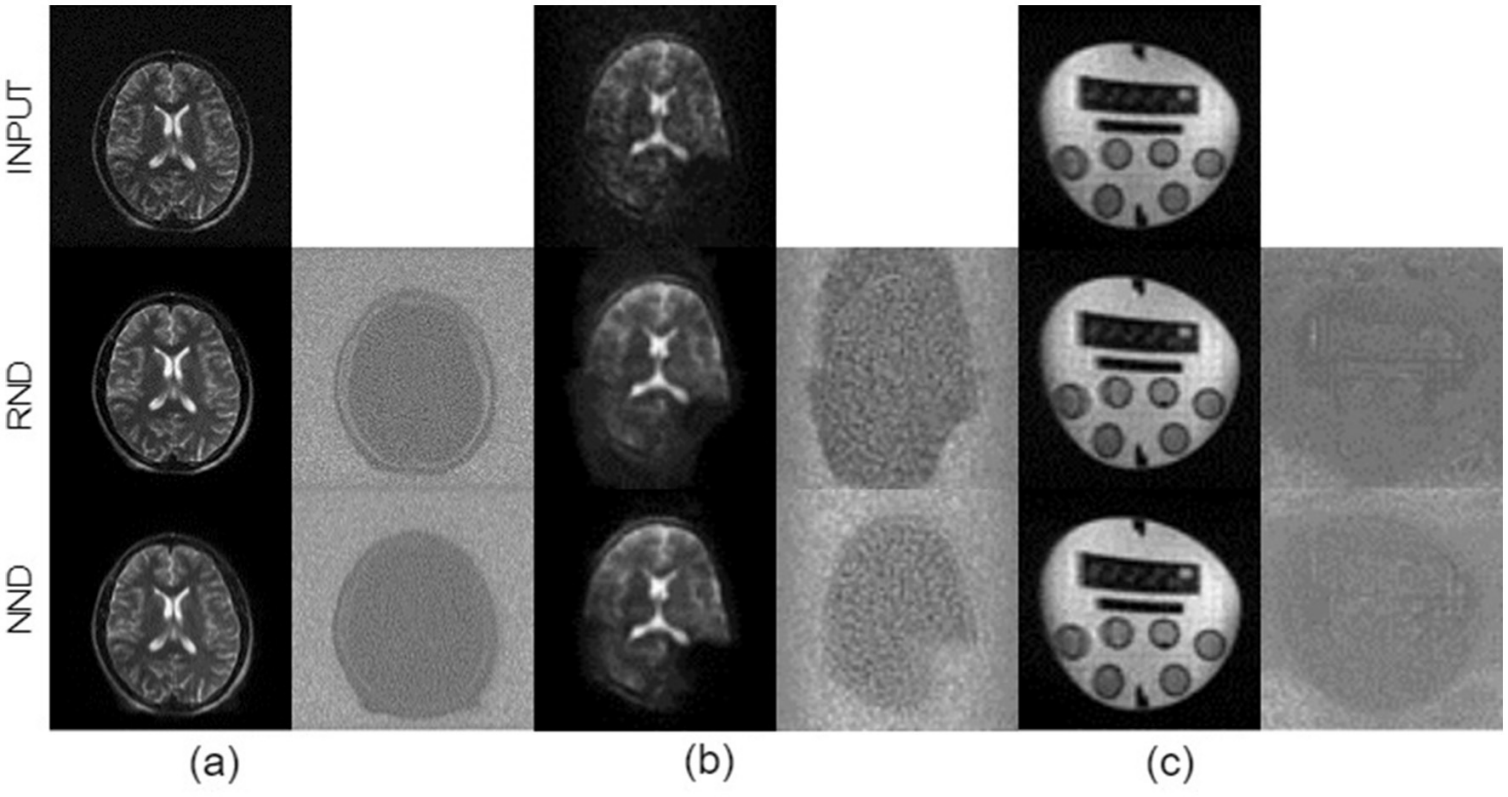
Comparison of denoising techniques across different MRI datasets for full images. The figure displays results from three datasets: **(a)** M4Raw, **(b)** *In vivo*, and **(c)** Phantom. Each dataset includes the Input image (top row), followed by outputs from the Random noise Denoising (RND, middle row) and the Native noise Denoising (NND, bottom row). The figure also depicts their respective difference maps to highlight the denoising efficacy.

**Figure 9:**
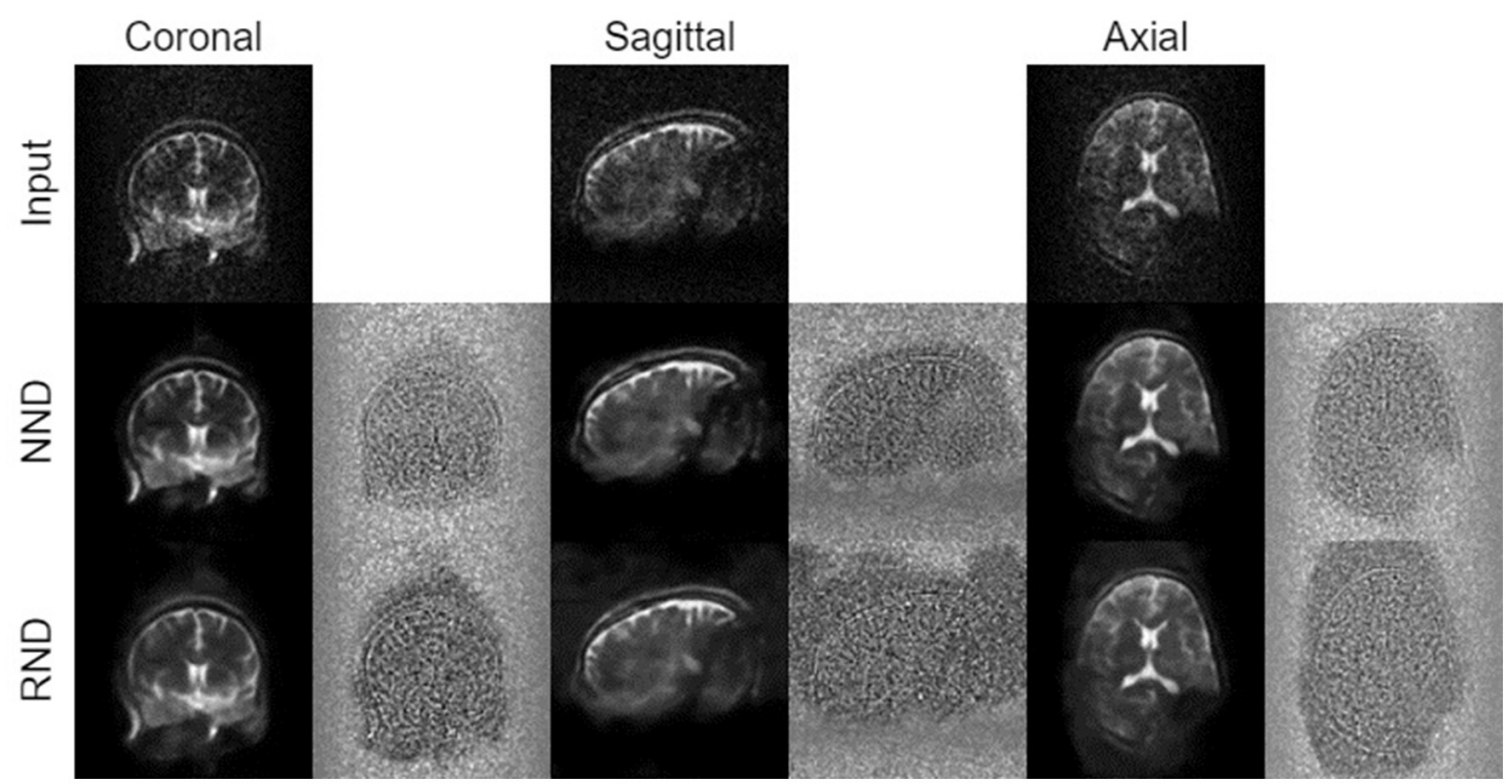
Out-of-distribution evaluation: the model trained on *in vivo* axial noise was evaluated on sagittal and coronal images. The difference maps indicated NND's **(bottom row)** superiority over RND **(top row)** in preserving brain structure across all views distinctly evident around the edges.

Qualitative assessment of the models shows the superiority of NND over RND in preserving image structure across all three datasets, as seen in Figures 7, 8. Difference maps (Figure 7) indicate that NND maintains sharper tissue boundaries while effectively reducing noise, particularly at the white-gray matter interface. The line intensity plots further confirm that NND preserves steep gradient transitions at tissue boundaries, whereas RND tends to smooth these transitions, potentially leading to structural information loss. Additionally, the effective receptive field (ERF) analysis (Figure 7) shows that NND activates more distinctly along anatomical structures, whereas RND exhibits diffuse activations, further demonstrating its advantage in structural preservation. Furthermore, qualitative evaluation of NND across individual plane views of the vivo dataset further highlights the superiority of NND over RND (see Figure 9) where we see vague boundaries for RND as compared to NND, with RND yielding utterly hazy boundaries for the sagittal view.

**Figure 10:**
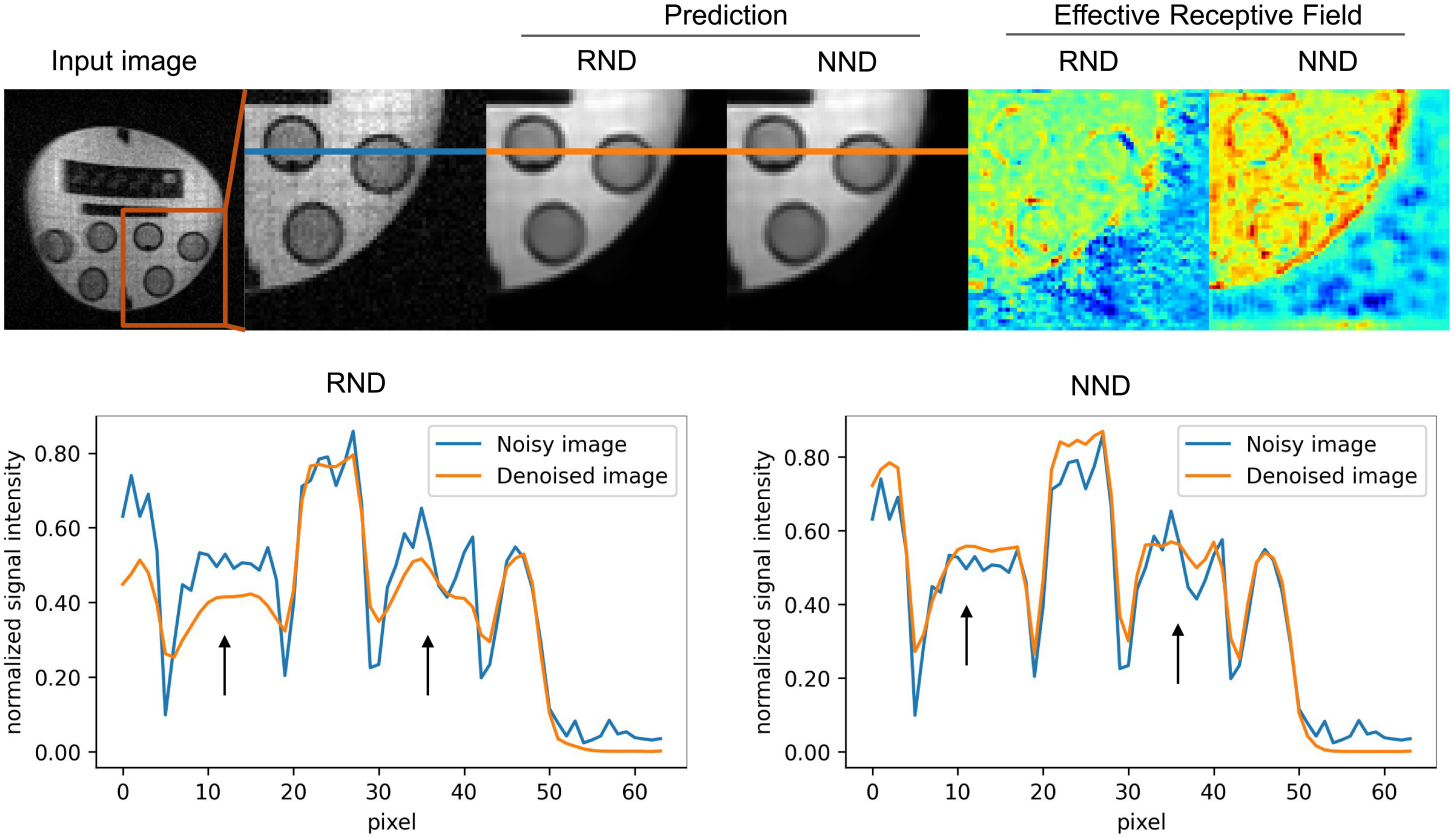
Edge preservation assessment: the model trained with noise from *in vivo* data is evaluated on phantom data from the same scanner. The results in the image above show a comparison between native noise denoising (NND) and random noise denoising (RND). In the top row, zoomed regions from a sample image demonstrate edge preservation for both NND and RND after model prediction. Comparatively, NND shows better edge preservation than RND, as evidenced by the effective receptive fields. In the bottom row, line intensity plots across the noisy patch (blue line) and corresponding patches from the predictions (orange line) for both NND and RND further show that NND yields sharper edge roll-offs (black arrows).

We further evaluated the models trained with noise from the *in vivo* dataset, on the phantom dataset to qualitatively assess how well they would preserve structure in the images. It can be seen from Figure 10 that the model distinctly activates for the background including within the phantom itself. It can also be seen that even when the mesh in the phantom is marred by noise, NND still manages to activate in such a way that the background mesh structure is preserved while greatly reducing the noise in the surrounding regions. From the line intensity plots in Figure 10, it can be seen that NND achieves sharper cut-offs at the edges compared to RND where the transition between the image and the background is more gradual. Such slight differences in gradient can result in rather significant differences in perceived image quality.

## 4 Discussions and conclusions

This study extends the application of native noise modeling in denoising low-field MRI images. We evaluated the native noise denoising (NND) approach across three datasets: the 0.3T M4Raw dataset, 0.05T *in vivo* brain MRI, and 0.05T phantom images. Our results demonstrate that the NND approach generally outperforms random noise denoising (RND), achieving statistically significant SNR improvements of 32.76%,19.02%, and 8.16% for the M4Raw, *in vivo* and phantom datasets respectively. Qualitative assessments further confirm NND's superior capability in preserving structural details and edges, particularly evident in the effective receptive fields and the difference maps analysis (Figures 7, 8).

While conventional denoising approaches for low-field MRI (< 0.1*T*) primarily rely on Gaussian noise assumptions, our work demonstrates the advantages of modeling the inherent Rician distribution characteristic of single-coil acquisitions (Figure 1). This approach addresses a critical gap in current methods, where traditional Gaussian-based denoising techniques often fail to account for the unique noise characteristics of very low-field systems. Our results indicate the importance of accurate noise modeling in improving image quality, particularly in systems where SNR is inherently limited (Tables 2, 3)

However, our approach has several important limitations that have to be considered. First, we assume minimal EMI noise, which may not reflect various real-world imaging environments, particularly in settings without magnetic shielding. Second, the scarcity of large low-field MRI datasets compared to high-field MRI (*B*_0_≥1.5*T*) limits comprehensive validation. Third, our evaluation currently lacks pathological cases, as we have focused primarily on healthy subjects and phantom data. This limitation is particularly significant for clinical translation because pathological conditions can present unique imaging challenges–lesions, tumors, and other abnormalities may alter local tissue contrast and introduce additional complexity to the denoising task. The absence of such cases in our validation means that we cannot yet guarantee NND's performance in preserving subtle pathological features that could be critical for diagnosis. In addition, different pathologies may present varying noise characteristics that our current model is not trained to handle.

Building on these initial findings, our current work focuses on evaluating NND across multiple neural network architectures (including transformers, GANs, and ResNets) to validate that the benefits of native noise modeling are architecture-independent and to identify optimal architectures for different clinical scenarios. We are expanding our evaluation to include a larger cohort of *in vivo* subjects–both healthy volunteers and patients–and extending the application of NND to other anatomical regions beyond the brain, such as the knee. These ongoing studies aim to refine our denoising approach and confirm its robustness and versatility.

Future work will involve integrating NND into real-time image reconstruction pipelines and assessing its clinical utility in multi-site settings, particularly in resource-limited regions. Our current established collaborations with a newly established MRI research laboratory in Uganda and Bangladesh will facilitate the deployment of these advancements, ultimately improving the accessibility and quality of low-field MRI for underserved populations.

## Data Availability

The original contributions presented in the study are included in the article/supplementary material, further inquiries can be directed to the corresponding authors.

## References

[B1] Abadi M. Agarwal A. Barham P. Brevdo E. Chen Z. Citro C.. (2015). TensorFlow: Large-scale machine learning on heterogeneous systems. Available online at: tensorflow.org

[B2] Aggarwal K. Cong Y. Lee J. H. Mani V. Calcagno C. Holbrook M. R.. (2023). “Feasibility of textural analysis and very-low-field magnetic resonance for imaging Nipah virus infection,” in ISMRM Conference Proceeding.

[B3] Aggarwal P. P. K. Jimeno M. M. Geethanath S. (2023). Repeatability of image quality in very low field MRI. arXiv:2304.07267. 10.48550/arXiv.2304.07267 38840502

[B4] Anazodo U. C. Ng J. J. Ehiogu B. Obungoloch J. Fatade A. Mutsaerts H. J. M. M.. (2023). A framework for advancing sustainable magnetic resonance imaging access in africa. NMR Biomed. 36:e4846. 10.1002/nbm.4846 36259628

[B5] Arnold T. C. Freeman C. W. Litt B. Stein J. M. (2023). Low-field MRI: clinical promise and challenges. J. Magn. Reson. Imaging 57, 25–44. 10.1002/jmri.28408 36120962 PMC9771987

[B6] Bian W. Li P. Zheng M. Wang C. Li A. Li Y.. (2024). “A review of electromagnetic elimination methods for low-field portable MRI scanner,” in 2024 5th International Conference on Machine Learning and Computer Application (ICMLCA) (New York, NY: IEEE), 614–618. 10.1109/ICMLCA63499.2024.10753737

[B7] Bisong E. (2019). “Google colaboratory,” in Building Machine Learning and Deep Learning Models on Google Cloud Platform: A Comprehensive Guide for Beginners, ed. E. Bisong (Berkeley, CA: Apress), 59–64. 10.1007/978-1-4842-4470-8_7

[B8] Chaudhari A. Kulkarni J. (2021). “Noise estimation in single coil MR images,” in 2021 IEEE International Conference on Imaging Systems and Techniques (IEEE), 100017. 10.1016/j.bea.2021.100017

[B9] Dougherty G. (2009). Digital Image Processing for Medical Applications. Cambridge: Cambridge University Press. 10.1017/CBO9780511609657

[B10] El-Shafai W. El-Nabi S. A. Ali A. M. El-Rabaie E.-S. M. El-Samie F. E. A. (2023). Traditional and deep-learning-based denoising methods for medical images. Multimed. Tools Appl. 83, 52061–52088. 10.1007/s11042-023-14328-x

[B11] Geethanath S. Poojar P. Ravi K. S. Ogbole G. (2021). MRI Denoising Using Native Noise. ISMRM and SMRT Annual Meeting and Exhibition.

[B12] Geethanath S. Vaughan J. T. Jr. (2019). Accessible magnetic resonance imaging: a review. J. Magn. Reson. Imaging 49, e65–e77. 10.1002/jmri.26638 30637891

[B13] Goodfellow I. Bengio Y. Courville A. (2016). Deep Learning. Cambridge: MIT Press. Available online at: http://www.deeplearningbook.org (accessed October 22, 2016).

[B14] Gudbjartsson H. Patz S. (1995). The rician distribution of noisy MRI data. Magn. Reson. Med. 34, 910–914. 10.1002/mrm.1910340618 8598820 PMC2254141

[B15] Horé A. Ziou D. (2010). “Image quality metrics: PSNR vs. SSIM,” in 2010 20th International Conference on Pattern Recognition (New York, NY: IEEE), 2366–2369. 10.1109/ICPR.2010.579

[B16] Kingma D. P. Ba J. (2017). Adam: a method for stochastic optimization. arXiv. 10.48550/arXiv.1412.6980

[B17] Koonjoo N. Zhu B. Bagnall G. C. Bhutto D. Rosen M. S. (2021). Boosting the signal-to-noise of low-field MRI with deep learning image reconstruction. Sci. Rep. 11:8248. 10.1038/s41598-021-87482-7 33859218 PMC8050246

[B18] Le D. B. T. Sadinski M. Nacev A. Narayanan R. Kumar D. (2021). “Deep learning–based method for denoising and image enhancement in low-field MRI,” in 2021 IEEE International Conference on Imaging Systems and Techniques (IST) (IEEE), 1–6. 10.1109/IST50367.2021.9651441

[B19] Lee J.-G. Jun S. Cho Y.-W. Lee H. Kim G. B. Seo J. B.. (2017). Deep learning in medical imaging: general overview. Korean J. Radiol. 18, 570–584. 10.3348/kjr.2017.18.4.570 28670152 PMC5447633

[B20] Lyu M. Mei L. Huang S. Zhu H. Zhang M. (2023). M4raw: a multi-contrast, multi-repetition, multi-channel MRI k-space dataset for low-field MRI research. Sci. Data 10:264. 10.1038/s41597-023-02181-4 37164976 PMC10172399

[B21] Marques J. P. Simonis F. F. Webb A. G. (2019). Low-field MRI: an MR physics perspective. J. Magn. Reson. Imaging 49, 1528–1542. 10.1002/jmri.26637 30637943 PMC6590434

[B22] Ronneberger O. Fischer P. Brox T. (2015). “U-net: convolutional networks for biomedical image segmentation,” in Medical Image Computing and Computer-Assisted Intervention – *MICCAI 2015*, eds. N. Navab, J. Hornegger, W. M. Wells, and A. F. Frangi (Springer International Publishing: New York), 234–241. 10.1007/978-3-319-24574-4_28

[B23] Saladi S. Prabha N. A. (2017). Analysis of denoising filters on mri brain images. Int. J. Imaging Syst. Technol. 27, 201–208. 10.1002/ima.22225

[B24] Shedbalkar S. Bhat S. G. Padiyar S. P. Murali S. Gunjir V. A. Sandeep K. B.. (2021). Comparative evaluation of image denoising techniques for magnetic resonance imaging. J. Magn. Reson. 25, 1836–1845.

[B25] Suhas S. Venugopal C. R. (2017). “MRI image preprocessing and noise removal technique using linear and nonlinear filters,” in 2017 IEEE International Conference on Imaging Systems and Techniques (IST) (Beijing: IEEE), 1–4. 10.1109/ICEECCOT.2017.8284595

[B26] Tong T. Caballero J. Bhatia K. Rueckert D. (2016). “Dictionary learning for medical image denoising, reconstruction, and segmentation,” in Machine Learning and Medical Imaging, The Elsevier and MICCAI Society Book Series, eds. G. Wu, D. Shen, and D. Terzopoulos (London: Academic Press), 153–181. 10.1016/B978-0-12-804076-8.00006-2

[B27] Vega F. Addeh A. MacDonald M. E. (2023). Denoising simulated low-field MRI (70mT) using denoising autoencoders (DAE) and cycle-consistent generative adversarial networks (cycle-GAN). arXiv:2307.06338. 10.48550/arXiv.2307.06338

[B28] Zbontar J. Knoll F. Sriram A. Murrell T. Huang Z. Muckley M. J.. (2019). fastMRI: An open dataset and benchmarks for accelerated MRI. arXiv [Preprint]. Available online at: http://arxiv.org/abs/1811.08839 (accessed April 22, 2025).

[B29] Zhang K. Zuo W. Chen Y. Meng D. Zhang L. (2017). Beyond a gaussian denoiser: residual learning of deep cnn for image denoising. IEEE Trans. Image Process. 26, 3142–3155. 10.1109/TIP.2017.2662206 28166495

